# Painful nerve injury increases plasma membrane Ca^2+^-ATPase activity in axotomized sensory neurons

**DOI:** 10.1186/1744-8069-8-46

**Published:** 2012-06-19

**Authors:** Geza Gemes, Katherine D Oyster, Bin Pan, Hsiang-En Wu, Madhavi Latha Yadav Bangaru, Qingbo Tang, Quinn H Hogan

**Affiliations:** 1 Medical College of Wisconsin, Department of Anesthesiology, 8701 Watertown Plank Road, Milwaukee, WI 53226, USA; 2 Department of Anesthesiology and Intensive Care Medicine, Medical University of Graz, Auenbruggerplatz 29, 8036 Graz, Austria; 3 Zablocki VA Medical Center, 5000 W. National Avenue, Milwaukee, WI 53295, USA

**Keywords:** PMCA, Dorsal root ganglion, Neuron, Calcium, Nerve injury

## Abstract

**Background:**

The plasma membrane Ca^2+^-ATPase (PMCA) is the principal means by which sensory neurons expel Ca^2+^ and thereby regulate the concentration of cytoplasmic Ca^2+^ and the processes controlled by this critical second messenger. We have previously found that painful nerve injury decreases resting cytoplasmic Ca^2+^ levels and activity-induced cytoplasmic Ca^2+^ accumulation in axotomized sensory neurons. Here we examine the contribution of PMCA after nerve injury in a rat model of neuropathic pain.

**Results:**

PMCA function was isolated in dissociated sensory neurons by blocking intracellular Ca^2+^ sequestration with thapsigargin, and cytoplasmic Ca^2+^ concentration was recorded with Fura-2 fluorometry. Compared to control neurons, the rate at which depolarization-induced Ca^2+^ transients resolved was increased in axotomized neurons after spinal nerve ligation, indicating accelerated PMCA function. Electrophysiological recordings showed that blockade of PMCA by vanadate prolonged the action potential afterhyperpolarization, and also decreased the rate at which neurons could fire repetitively.

**Conclusion:**

We found that PMCA function is elevated in axotomized sensory neurons, which contributes to neuronal hyperexcitability. Accelerated PMCA function in the primary sensory neuron may contribute to the generation of neuropathic pain, and thus its modulation could provide a new pathway for peripheral treatment of post-traumatic neuropathic pain.

## Background

Influx of Ca^2+^, the dominant second messenger in neurons, follows neuronal activation by membrane depolarization or ligand binding. Although much of this Ca^2+^ is initially buffered and sequestered in intracellular stores, including the endoplasmic reticulum and mitochondria, it must ultimately be discharged from the cell. The principal pathways for this process are the Na^+^/Ca^2+^ exchanger (NCX), which has high transport capacity but low affinity [1], and the plasma membrane Ca^2+^-ATPase (PMCA), which has high affinity for Ca^2+^ (200nM) but low capacity. For sensory neurons, PMCA is the dominant extrusion pathway for Ca^2+^ that enters during low amplitude transients (peak [Ca^2+^_c_ <400nM) [2,3] such as those that accompany typical patterns of activation [4], PMCAs, predominantly the 2a and 4b isoforms in sensory neurons [5], generate Ca^2+^ efflux against an approximately 2 × 10^5^-fold gradient through the consumption of ATP, with an obligatory influx of hydrogen ions from the extracellular space.

Calcium signals in neurons control critical functions including differentiation, growth, excitability, synaptic transmission, cytotoxicity and apoptosis. We have previously found that painful peripheral nerve injury is accompanied by disordered Ca^2+^ signaling in the traumatized sensory neurons of the dorsal root ganglion (DRG). Specifically, neurons axotomized by spinal nerve ligation (SNL) develop a decreased resting [Ca^2+^_c_[6]. This level is set by the balance of Ca^2+^ influx regulated by levels of stored Ca^2+^, so-called store-operated Ca^2+^ entry (SOCE) versus Ca^2+^ discharge by PMCA [7,8]. Our observation that SOCE is amplified by axotomy in sensory neurons [7] therefore suggests that PMCA may be activated by injury. Furthermore, we have identified more rapid resolution of depolarization-induced transients after injury [9], which additionally implicates upregulation of PMCA. Finally, other research has shown that PMCA action reduces the size of the afterhyperpolarization (AHP) that follows action potentials (APs) [5,10], possibly leading to elevated neuronal excitability. Reduced AHP and neuronal hyperexcitability are features of axotomized neurons after SNL [11], so these findings together suggest a possible role of PMCA in the pathogenesis of neuropathic pain.

To characterize the effect of nerve injury, we have measured PMCA function in rats subjected to SNL. This injury creates fully axotomized neurons in the 5^th^ lumbar (L5) DRG. In contrast, the neurons of L4 remain intact but are exposed to inflammatory conditions in the distal sciatic nerve triggered by degenerating distal fragments of axotomized L5 neurons. Accordingly, we have evaluated these populations separately and compared them to control neurons from animals receiving only skin incision. The influence of PMCA upon excitability has not been directly examined, so we additionally evaluated this critical physiological role.

## Results

Rats were subjected to spinal nerve ligation or anesthesia and skin incision only (control group) and hyperalgesic behavior was assessed at three different time points by the response to mechanical stimulation with a 22 G spinal needle. Hyperalgesia-type responses developed in SNL rats (44 ± 10%, n = 14) but not in control rats (0 ± 0%, n = 20; *P* < 0.001). The accuracy of the SNL surgery was confirmed at the time of tissue harvest in all SNL animals.

### Identification of PMCA function

**Figure 1:**
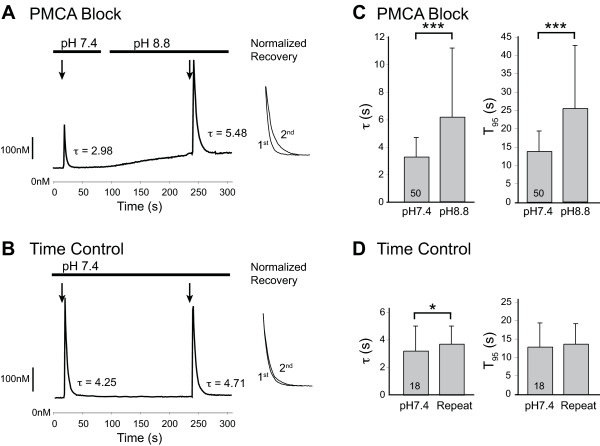
**PMCA regulates [Ca**^**2+**^**]**_**c**_** in sensory neurons at rest and after activation.** Sample traces demonstrate that blocking PMCA by switching from bath solution with pH 7.4 to pH 8.8 increases resting [Ca^2+^]_c_ levels (**A**), whereas time alone has no effect (**B**). Transients induced by application of high K^+^ solution (50 mM for 0.3 s, arrows) are prolonged by pH 8.8, as measured by recovery rate constant (τ) and by the time to achieve 95% recovery to baseline (T_95_), as shown in the sample traces (**A**) and summary data (**C**). Time alone minimally prolongs τ but not T_95_ (**B**, **D**). Insets in panels **A** and **B** show superimposed recovery traces from the first and second depolarizations after normalization to identical amplitudes. Bar graphs show mean ± SD; numbers in bars indicate n; * *P* < 0.05, *** *P* < 0.001, by paired *T*-test.

To characterize the role of PMCA in normal and injured neurons, we first evaluated whether PMCA is active in control neurons at rest. Ca^2+^ extrusion by PMCA requires the influx of hydrogen ions, and elevated extracellular pH can block the function of PMCA [2], with a peak effect at pH 8.8 [12], In acutely dissociated DRG neurons, we noted a progressive rise of [Ca^2+^_c_ (30 ± 41nM over 3 min, n = 50; Figure 1A) upon changing the bath solution to pH 8.8, whereas resting [Ca^2+^_c_ changed minimally in time controls (5 ± 4nM, n = 18, *P* < 0.01 vs. pH 8.8; Figure 1B). This indicates constitutive operation of the PMCA that contributes to the regulation of Ca^2+^ levels in resting sensory neurons. When the neurons were activated by depolarization by a 0.3 s K^+^ stimulus, blockade of PMCA by pH8.8 resulted in prolongation of the Ca^2+^ transient (increased τ and T_95_) compared to a prior depolarization in pH7.4 bath (Figure 1A, C). Time controls in pH7.4 also showed increased τ at the second depolarization (Figure 1B, D), but this increase was much smaller (23 ± 19%) than that produced by switching to pH8.8 (83 ± 99%, *P* < 0.05).

**Figure 2:**
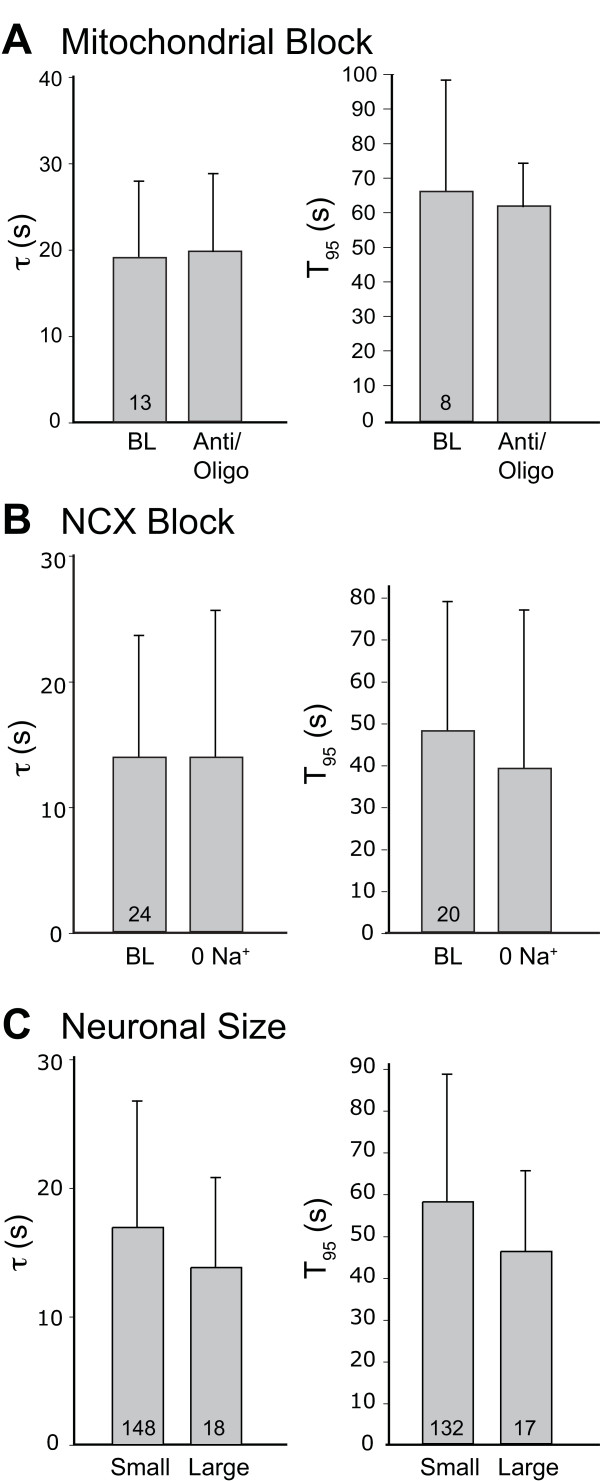
**Roles of mitochondria, Na**^**+**^**/Ca**^**2+**^** exchanger (NCX), and neuronal size.** The recovery of [Ca^2+^]_c_ from transients induced by application of high K^+^ solution (50 mM for 0.3 s) in thapsigargin-treated neurons was measured by recovery rate constant (τ) and by the time to achieve 95% recovery to baseline (T_95_). Blockade of mitochondrial function by combined application of antimycin (1 μM) and oligomycin (10 μM) for 3 min had no effect compared baseline values (BL) in the same neurons (**A**). Block of NCX by equimolar replacement of bath Na^+^ with N-methyl-d-glucamine (NMDG) also had no effect on recovery of transients (**B**). Neuronal groups with either large (diameter 39 ± 4 μm) or small (26 ± 3 μm) profiles did not differ in PMCA function (**C**). Mean ± SD; numbers in bars indicate n; no differences when tested by paired *T*-test (**A**, **B**) and simple *T*-test (**C**).

These observations reveal the participation of PMCA in regulating Ca^2+^ signaling after neuronal activation. However, the simultaneous operation of other clearance mechanisms precludes the use of this approach for the measurement of the specific activity of PMCA *per se* under different injury conditions. We therefore employed the strategy of measuring PMCA selectively after eliminating the function of other Ca^2+^ sequestration pathways. First, we blocked sarcoplasmic-endoplasmic reticulum Ca^2+^ ATPase (SERCA), which pumps cytoplasmic Ca^2+^ into the ER, by exposure to thapsigargin (TG, 1 μM, 5 min). This resulted in transients that resolved more slowly than in the absence of TG (Figure 2), which indicates that SERCA assists PMCA in clearing activity-induced Ca^2+^ loads. Additionally, by using brief depolarizations, we limited transient amplitude to levels (400nM) that are insufficient to initiate mitochondrial buffering of cytoplasmic Ca^2+^[5]. Finally, traces that showed a shoulder or plateau of sustained Ca^2+^ elevation during the descending limb of the activity-induced transient were not included in the evaluation of PMCA, as this pattern represents the participation of mitochondrial buffering [13]. These two criteria required exclusion of 25% of neurons.

To test if any residual mitochondrial Ca^2+^ uptake persisted under these conditions, we examined depolarization-induced transients in TG-treated neurons for sensitivity to mitochondrial blockers. Although the protonophore carbonyl cyanide-p-trifluoromethoxyphenylhydrazone (FCCP) prolonged transients, this uncoupling of oxidative phosphorylation also leads to robust hydrolysis of cytoplasmic ATP [14], and thereby inhibits PMCA function. As an alternative approach, we therefore employed the combined application of inhibitors of mitochondrial ATP synthase (oligomycin, 10 μM) and mitochondrial electron transport (antimycin, 1 μM), thereby eliminating mitochondrial Ca^2+^ uptake without affecting ATP levels [14]. These agents produced no effect on the rate of Ca^2+^ transient recovery (Figure 2A), which provides assurance that activity-induced transients under our baseline conditions do not engage the Ca^2+^ buffering function of mitochondria.

NCX contributes minimally to Ca^2+^ extrusion after neuronal activation in adult [2] and neonatal [5] sensory neurons during patch recording. We examined NCX in intact adult sensory neurons by replacement of bath Na^+^ with N-methyl-D-glucamine (NMDG), which similarly did not alter the Ca^2+^ recovery kinetics of transients in TG-treated neurons (Figure 2B). An effect of NCX on resting [Ca^2+^_c_ has been noted in DRG neurons during patch recording [15], but such changes were not noted in our recordings (data not shown).

**Figure 3:**
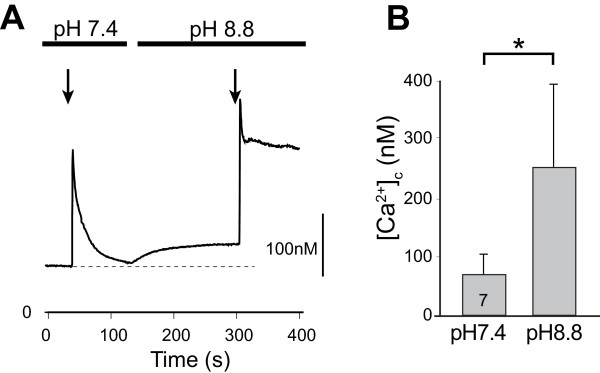
**Blockade of PMCA in thapsigargin-treated neurons.** Changing bath solution to pH 8.8 produced an increase in resting [Ca^2+^]_c_ (**A**, **B**), and prevented recovery of transients (**A**) in neurons following blockade of SERCA with thapsigargin (100nM). Mean ± SD; number in bar indicates n; * *P* < 0.05 by paired *T*-test.

On the basis of these initial observations, we adopted the approach of measuring PMCA function by determining the rate of recovery of small, depolarization-induced transients in TG-treated sensory neurons. To confirm that this successfully isolates PMCA as the only functioning mechanism for clearing Ca^2+^ from the cytoplasm, we compared the recovery from small activation-induced Ca^2+^ loads in TG-treated neurons before and after switching the bath pH to 8.8 (Figure 3A). Application of pH 8.8 solution substantially increased resting [Ca^2+^_c_ (Figure 3B), reflecting unopposed Ca^2+^ entry through store-operated Ca^2+^ channels [7,8]. Transients induced by K^+^ depolarization did not recover in any neurons (n = 7; Figure 3A), which demonstrates that PMCA is the only available clearance pathway following test depolarizations under our study conditions.

Sensory neurons are a diverse population. For instance, neurons with small somatic area generally convey nociceptive traffic, while low threshold stimuli activate larger neurons [16]. We examined whether PMCA function differed in resting sensory neurons of different sizes (Figure 2C), but found no significant difference in the rate of recovery from Ca^2+^ loads.

### Effect of injury on PMCA

**Figure 4:**
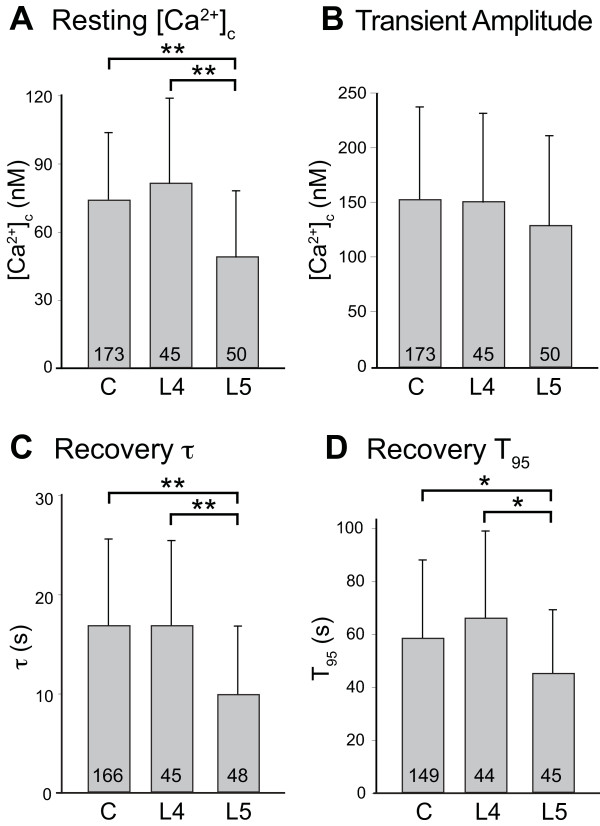
**Effects of neuronal injury on resting [Ca**^**2+**^**]**_**c**_** and activity-induced transients.** Neurons harvested from the 4^th^ lumbar (L4) and L5 dorsal root ganglia after spinal nerve ligation were compared to control neurons (C). Injury in the L5 group decreased resting [Ca^2+^]_c_ (**A**) but not transient amplitude (**B**). Recovery of transients induced by application of high K^+^ solution (50 mM for 0.3 s) was accelerated in the L5 population after injury, measured by both the recovery rate constant (τ) and by the time to achieve 95% recovery to baseline (T_95_). Mean ± SD; numbers in bars indicate n; * *P* < 0.05, ** *P* < 0.01 by ANOVA with Bonferroni’s *post hoc* test.

Following TG treatment, resting [Ca^2+^_c_ was depressed in axotomized neurons isolated from the L5 DRG after SNL compared to control neurons (Figure 4A). We have previously observed a similar injury-induced decrease in [Ca^2+^_c_ in the absence of TG treatment [6]. These findings jointly indicate that injury reduces resting [Ca^2+^_c_ by affecting PMCA function. The current data do not confirm our previous finding that injury reduces transient amplitude (Figure 4B) [9]. However, depolarizations in the present study were more brief, and we specifically excluded transients over 400nM, which may have reduced the apparent effect of injury on transient amplitude.

Comparison of PMCA function in axotomized SNL L5 neurons and neighboring SNL L4 neurons to control neurons (treated with TG) revealed an activated PMCA state selectively in axotomized neurons. Specifically, measurement of both τ (Figure 4C) and T_95_ (Figure 4D) revealed an accelerated recovery of the depolarization-induced transient in the SNL L5 population. PMCA activity is regulated by the concentration of Ca^2+^ in the cytoplasm [17]. The diameter of the axotomized SNL L5 neurons (24.4 ± 3.3 μm, n = 50) was smaller than both the control (27.5 ± 5.1 μm, n = 173, *P* < 0.001) and the SNL L4 neurons (29.4 ± 5.5 μm, n = 45, *P* < 0.001), consistent with prior observations on the effects of sensory neuron axotomy [11,18]. To eliminate a possible influence of neuronal size *per se*, we examined PMCA function in neuronal populations with diameters between 23 μm and 29 μm (average for Control 26.2 ± 18 μm, n = 103; SNL L4 26.3 ± 1.6, n = 29; SNL L5 25.7 ± 1.1, n = 20). In these restricted populations, the τ for transient recovery was still significantly depressed in the SNL L5 neurons (10.2 ± 8.5 s) compared to the SNL L4 (16.3 ± 9.6 s) and control neurons (16.9 ± 9.1 s; *P* < 0.05). This indicates that injury, rather than a shift in neuronal size, activates PMCA after axotomy.

**Figure 5:**
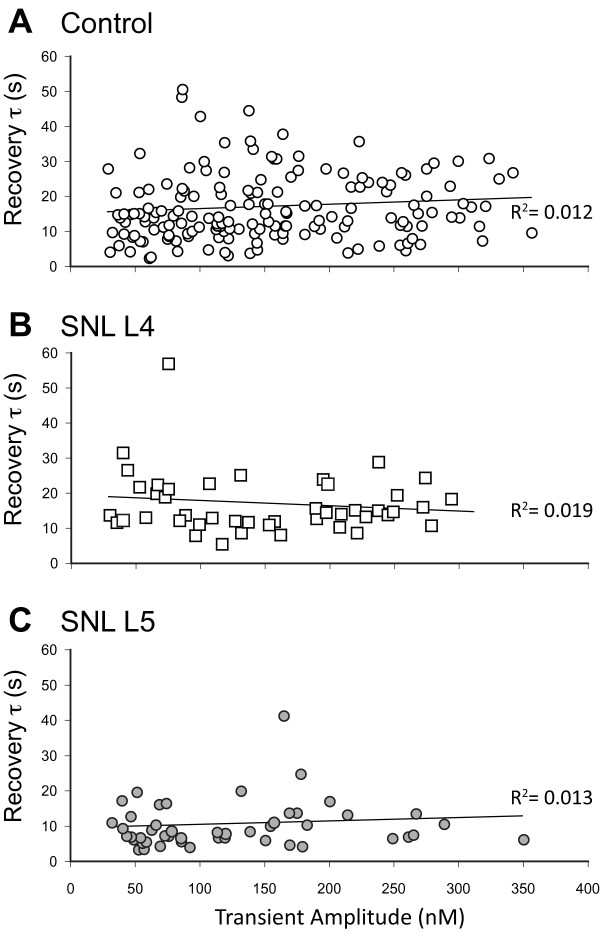
**Transient amplitude does not influence PMCA function.** The recovery of [Ca^2+^]_c_ from transients induced by application of high K^+^ solution (50 mM for 0.3 s) in thapsigargin-treated neurons was measured by recovery rate constant (τ) in control neurons (**A**) and neurons from the 4^th^ lumbar (L4, **B**) and L5 dorsal root ganglia (**C**) after spinal nerve ligation (SNL). In each, transient amplitude did not influence τ (regression *P* of 0.16, 0.36, and 0.43 respectively). The best-fit trend line and coefficient of determination (R^2^) are shown.

Since injury depresses Ca^2+^ entry through voltage-gated Ca^2+^ channels [19], we examined whether the amount of Ca^2+^ accumulation during activation may itself affect PMCA function. Regression analysis showed no influence of transient amplitude upon τ (Figure 5), which supports a direct effect of injury on PMCA independent of the level of Ca^2+^ influx. To further examine whether the reduced transient amplitude in injured neurons affects recovery, we compared recovery kinetics in the different injury groups using only transients with amplitudes between 50 and 200nM. Limited this way, groups showed comparable average amplitudes (Control 118 ± 40nM, n = 103; SNL L4 121 ± 48nM, n = 26; SNL L5 118 ± 46 nM, n = 36), but SNL L5 neurons still had more rapid PMCA function (τ of 10.2 ± 7.5 s) compared to Control (16.9 ± 10.2 s, *P* < 0.01) or SNL L4 (16.8 ± 9.9 s, *P* < 0.05). These findings strongly indicate that accelerated function of PMCA after injury is due to alteration of the pump and not a result of loading conditions.

### Influence of neuronal activation on PMCA

**Figure 6:**
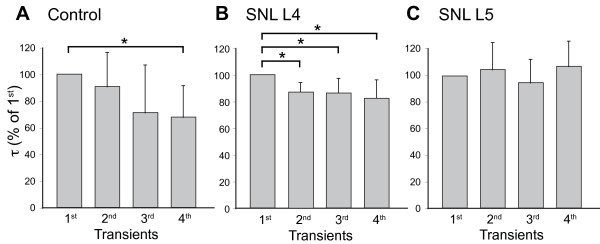
**Influence of neuronal activity on PMCA function.** The rate constant (τ) for recovery was determined for transients induced at 2 min intervals by application of high K^+^ solution (50 mM for 0.3 s) in thapsigargin-treated neurons (n = 6). Repeated activation caused progressive acceleration of the recovery in control neurons (**A**) and neurons from the fourth lumbar dorsal root ganglia after spinal nerve ligation (SNL L4) (**B**), but not after axotomy in SNL L5 neurons (**C**). Mean ± SD; * *P* < 0.05 by repeated measures ANOVA with Dunnett’s *post hoc* test.

Axotomized neurons may be quiescent due to being disconnected from their receptive fields, or alternatively may be hyperactive from ectopically generated activity due to membrane instability or mechanical and inflammatory influences [20]. To identify whether PMCA function is sensitive to neuronal activity level, we exposed neurons to repeated Ca^2+^ loads similar to those we have previously recorded during AP trains conducted to the neuronal soma [4]. Specifically, we generated repeated 200-400nM Ca^2+^ transients in TG-treated sensory neurons by 0.3 s K^+^ depolarizations at 2 min intervals, and examined the effect of this activity on the rate of transient recovery (Figure 6A). This revealed a progressive acceleration of PMCA function in TG-treated control neurons, such that τ was decreased to 68 ± 12% of baseline after 3 preceding periods of activation (*P* < 0.05, n = 6), suggesting that neuronal hyperactivity could account for the increased PMCA function found in sensory neurons after nerve injury. Although activity-induced facilitation of PMCA function was also observed in neighboring L4 neurons after SNL (Figure 6B), axotomized SNL L5 neurons showed no acceleration of PMCA function (Figure 6C), which may indicate saturation of this form of plasticity after neuronal injury.

### Regulation of neuronal excitability by PMCA

**Figure 7:**
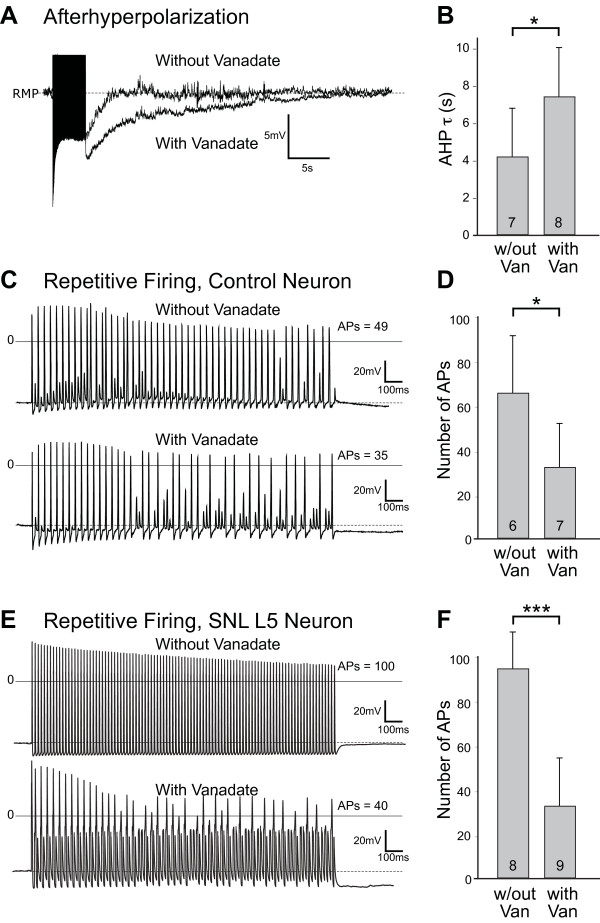
**Regulation of neuronal excitability by PMCA.** The afterhyperpolarization (AHP) that follows a train (50 Hz for 4 s) of action potentials (APs) generated by current injection (2x threshold) in control neurons are shown in sample traces (**A**) made by whole-cell patch-clamp recording 10 min after breakthrough, comparing a recording without vanadate to one in which the patch pipette contained vanadate (200 μM). The traces are aligned at their resting membrane potentials (RMP; -61 mV for the control neuron, -64 mV for the vanadate-treated neuron). The train of APs appears as a black band at the beginning of the trace with their amplitude truncated. Summary data (**B**) show that the duration of the AHP, measured as the time constant (τ) of a monoexponential fitted to the AHP, was prolonged by vanadate (Van) compared to other neurons without vanadate (w/out Van). The ability to generate APs was tested by determining the ability of a train (50 Hz for 2 s, total 100) of depolarizing stimuli (1.5x threshold) to generate APs. Sample traces in control neurons (**C**) show 49 full APs produced in a neuron without vanadate and 35 APs produced by comparable stimuli in a vanadate-treated neuron. Summary data (**D**) reveal a depression of AP generation by PMCA blockade with vanadate in control neurons. In neurons from the fifth lumbar dorsal root ganglia after spinal nerve ligation (SNL L5), sample traces (**E**) and summary data (**F**) show that the injury-induced difference in excitability is eliminated by vanadate treatment. Mean ± SD; numbers in bars indicate n; * *P* < 0.05, *** *P* < 0.001 by Mann Whitney test.

Since cytoplasmic Ca^2+^ regulates sensory neuron excitability [21,22], we speculated that PMCA function may modulate excitability through controlling the pace of Ca^2+^ extrusion from the cytoplasm after activity-induced influx. Electrophysiological studies were performed by patch technique in dissociated sensory neurons that did not differ between groups for diameter (30.5 ± 1.7 μm) or resting membrane potential (−60.7 ± 4.4 mV, n = 28). We first evaluated effects on the afterhyperpolarization (AHP), since this regulates repetitive firing behavior in sensory neurons [23,24]. We used intracellular vanadate, delivered by dialysis from the patch electrodes, to block PMCA function [2,5]. Vanadate also blocks SERCA function, so selective effects on PMCA were isolated by pretreatment with TG (1 μM). After allowing 10 min of dialysis for delivery of vanadate (Na^+^ orthovanadate, 200 μM) to the cytoplasm, trains of APs (50 Hz for 4 s) [10] were generated by stimulation at current amplitude of 2-fold above threshold, which assured AP generation for each stimulus (Figure 7A). Vanadate significantly prolonged the AHP, compared to separate control neurons in which vanadate was not included in the patch pipette (Figure 7B). This confirms prior findings in neonatal sensory neurons cultured for 2-3d [5].

The AHP and underlying channels are known to regulate neuronal excitability, particularly repetitive firing behavior [23,25]. To directly test if regulation of the AHP by PMCA affects excitability, we examined the ability of sensory neurons to generate APs during trains of depolarization stimuli with and without vanadate. Trains (50 Hz for 2 s) were generated in patched neurons (Figure 7C) by depolarizing stimuli at 1.5-fold above threshold (2.0 ± 0.2 nA, n = 6, for Control; 1.8 ± 0.4 nA, n = 7, for vanadate; *P* = 0.2). After 10 min of dialysis, AP generation was significantly less in neurons treated with vanadate (Figure 7D). Further evidence of an effect by vanadate is the observation in the vanadate group that the ability to generate trains of APs decreased 26 ± 27% after 10 min of dialysis compared to AP generation by an identical protocol immediately after breakthrough, whereas no change was evident during this time interval in the control group (−4 ± 4%, *P* < 0.05 vs. vanadate). These findings indicate that clearance of cytoplasmic Ca^2+^ by PMCA facilitates rapid firing in sensory neurons. Axotomized SNL L5 neurons stimulated by the same protocol without vanadate (50 Hz for 2 s, 1.5x threshold, 2.3 ± 0.4 nA, n = 8 Figure 7E) developed trains that on average contained more APs than control neurons, and included the maximum 100 APs in 7 of 8 cases. In other SNL L5 neurons (stimulus intensity 2.1 ± 0.4 nA, n = 9), vanadate reduced the number of APs (Figure 7F) to a level that was comparable to vanadate-treated control neurons. This suggests that a component of the elevated excitability of injured sensory neurons is attributable to their accelerated PMCA activity.

## Discussion

Prior studies by others have found that PMCA is a critical process by which sensory neurons clear Ca^2+^ from the cytoplasmic compartment after activity [2,3,5]. Since these studies used neurons from neonatal rats after prolonged culture (2-14d), their results may be specific to these conditions. For instance, animal age and duration of culture may affect the level of neuronal differentiation, which regulates PMCA expression [26]. Our findings extend these prior observations to neurons examined within hours following dissociation from adult animals, and confirm a key role of PMCA in the reestablishment of [Ca^2+^_c_ homeostasis following activity. Of particular interest, we have shown that painful peripheral nerve injury stimulates PMCA function in sensory neurons.

PMCA is generally thought to be the principal mechanism of Ca^2+^ extrusion from sensory neurons, although there have been conflicting reports regarding the relative contribution of the NCX to Ca^2+^ clearance in sensory neurons. While a contribution of NCX to Ca^2+^ extrusion from sensory neurons has been proposed by some studies [15,27], others have found no evidence of its participation [5,28,29], with which our findings agree. This supports the contention that after activation of sensory neurons, total cellular Ca^2+^ balance is reestablished predominantly by the action of PMCA.

The main finding of this investigation is that painful nerve injury results in increased PMCA function. In previous studies, we have demonstrated severe disruption of sensory neuron Ca^2+^ handling following axotomy, such as diminished Ca^2+^ influx through VGCCs during neuronal activation, decreased level of stored Ca^2+^ within the ER, and reduced [Ca^2+^_c_ in resting neurons. Since the dynamic balance between PMCA and SOCE establishes the resting [Ca^2+^_c_, our prior observation of increased Ca^2+^ entry through store-operated Ca^2+^ channels after injury [7] suggested that PMCA function must be elevated to an even greater degree. This is borne out by our present findings. We have also previously noted that activity-induced transients resolve more quickly following axonal injury [4,9], but the specific cause could not be ascertained without isolating selective Ca^2+^-handling components. The findings reported here indicate that accelerated PMCA function is at least one factor producing this effect. Although increased SERCA function could potentially contribute to more rapid resolution of transients following injury, our previous findings make this unlikely. Specifically, we have noted that injury produces a decreased intraluminal ER Ca^2+^ concentration while the constitutive leak of Ca^2+^ from the ER is unchanged [30], Together, these findings suggest that SERCA activity is diminished, but this has not been directly investigated.

There are several mechanisms by which PMCA function may be elevated following injury. Our data here indicate that a period of neuronal activity is followed by accelerated PMCA function. This resembles the conditioning effect previously noted in neonatal neurons after prolonged culture [31]. Following peripheral nerve injury, excessive neuronal firing through ectopic spontaneous activity [32,33] may be sufficient to condition PMCAs in sensory neuron somata. Neuronal activation may additionally lead elevated expression of PMCAs [34]. Finally, PMCAs are a regulatory target of kinase signaling, such as protein kinase C [5,35], protein kinase A [36], and protein tyrosine kinases [10]. These kinase pathways may be activated in sensory neurons by injury [37-39].

The physiological consequences of accelerated PMCA function in injured sensory neurons may be diverse. At the sensory neuron central synapse in the spinal dorsal horn, accelerated PMCA clearance of Ca^2+^ from the presynaptic terminal may diminish excitatory neurotransmission, as has been shown in the hippocampus [40]. This action would not be expected to contribute to hyperalgesia after nerve injury. However, in the peripheral portion of the sensory neuron, our present electrophysiological data show that suppression of PMCA with vanadate inhibits repetitive firing behavior, revealing a pro-excitatory regulatory role for PMCA function in the sensory neuron soma. Therefore, increased clearance of cytoplasmic Ca^2+^ by accelerated PMCA function after nerve injury will elevate neuronal excitability. Possible downstream mechanisms include diminished activation of Ca^2+^-activated K^+^ channels [24] and depressed CaMKII function [41]. Ectopic activity originates in the soma after peripheral nerve injury [33], so these impulse trains would be accelerated by the concurrent increased PMCA activity. A similar effect at the axonal T-junction, a site of impulse failure during high frequency afferent activity [42], may lead to elevated conduction rates for AP trains originating from sources in the periphery, such as neuromata [32]. These changes could all contribute to pain generation by increasing afferent neuronal traffic following peripheral injury. Finally, low resting [Ca^2+^_c_ may predispose neurons to the apoptosis [43] consistent with the progressive DRG neuron loss observed in the setting of chronic peripheral nerve injury [44].

## Conclusions

In this investigation, we found an upregulation of PMCA in adult DRG neurons after painful nerve injury. PMCA may be considered a novel site for altering mechanisms leading to the generation of neuropathic pain. The continuing development of molecular therapies and the tolerance of the DRG as a site for intraneuronal injections [45,46] make modulation of PMCA function in sensory neurons a potential future therapy for chronic pain.

## Methods

All methods and use of animals were approved by the Medical College of Wisconsin Institutional Animal Care and Use Committee.

### Injury model

Male Sprague–Dawley (Taconic Farms Inc., Hudson, NY) rats weighing 160 to 180 g were subjected to SNL modified from the original technique [47]. Specifically, rats were anesthetized with 2% isoflurane in oxygen and the right paravertebral region was exposed. The L6 transverse process was removed, after which the L5 and L6 spinal nerves were ligated with 6–0 silk suture and transected distal to the ligature. The muscular fascia was closed with 4–0 resorbable polyglactin suture and the skin closed with staples. Control animals received skin incision and closure only. After surgery, rats were returned to their cages and kept under normal housing conditions with access to pellet food and water *ad lib*.

### Sensory testing

Rats underwent sensory testing for a pattern of hyperalgesic behavior that we have previously documented to be associated with conditioned place avoidance [48,49]. Briefly, on 3 different days between 10d and 17d after surgery, right plantar skin was touched with a 22 G spinal needle with adequate pressure to indent but not penetrate the skin. Whereas control animals respond with only a brief reflexive withdrawal, rats following SNL may display a complex hyperalgesia response that includes licking, chewing, grooming and sustained elevation of the paw. The average frequency of hyperalgesia responses over the 3 testing days was tabulated for each rat.

### Neuron isolation and plating

Between 21d and 28d after surgery, ganglia from rats were rapidly harvested following isoflurane anesthesia and decapitation and were incubated in 0.01% blendzyme 2 (Roche Diagnostics, Indianapolis, IN) for 30 min followed by incubation in 0.25% trypsin (Sigma Aldrich, St. Louis, MO) and 0.125% DNAse (Sigma) for 30 min, both dissolved in Dulbecco´s modified Eagle´s medium (DMEM)/F12 with glutaMAX (Invitrogen, Carlsbad, CA). After exposure to 0.1% trypsin inhibitor and centrifugation, the pellet was gently triturated in culture medium containing Neural Basal Media A with B27 supplement (Invitrogen), 0.5 mM glutamine, 10 ng/ml nerve growth factor 7 S (Alomone Labs, Jerusalem, Israel) and 0.02 mg/ml gentamicin (Invitrogen). Dissociated neurons were plated onto poly-L-lysine coated glass cover slips (Deutsches Spiegelglas, Carolina Biological Supply, Burlington, NC) and maintained at 37°C in humidified 95% air and 5% CO_2_ for 2 hours, and were studied no later than 6 hours after harvest.

### Measurement of cytoplasmic Ca^2+^ concentration

Unless otherwise specified, the bath contained Tyrode´s solution (in mM): NaCl 140, KCl 4, CaCl_2_ 2, Glucose 10, MgCl_2_ 2, 4-(2-hydroxyethyl)-1-piperazineethanesulfonic acid (HEPES) 10, with an osmolarity of 297-300 mOsm and pH 7.40. Agents were obtained as follows: Fura-2-AM from Invitrogen, and thapsigargin (TG), antimycin and oligomycin from Sigma Aldrich. Stock solutions of TG and Fura-2-AM were dissolved in DMSO, and subsequently diluted in the relevant bath solution such that final bath concentration of DMSO was 0.2% or less, which does not effect [Ca^2+^]_c_ (n = 20, data not shown). The 500 μl recording chamber was superfused by gravity-driven flow at a rate of 3 ml/min. Agents were delivered by directed microperfusion controlled by a computerized valve system through a 500 μm diameter hollow quartz fiber 300 μm upstream from the neurons. This flow completely displaced the bath solution, and constant flow was maintained through this microperfusion pathway by delivery of bath solution when specific agents were not being administered. Dye imaging shows that solution changes were achieved within 200 ms.

Neurons plated on cover slips were exposed to Fura-2-AM (5 μM) at room temperature in a solution that contained 2% bovine albumin to aid dispersion of the fluorophore. After 30 min, they were washed 3 times with regular Tyrode’s solution, given 30 minutes for de-esterification, and then mounted in the recording chamber. Neurons were first examined under brightfield illumination, and those showing signs of lysis, crenulation or superimposed glial cells were excluded. For Ca^2+^ recording, the fluorophore was excited alternately with 340 nm and 380 nm wavelength illumination (150 W Xenon, Lambda DG-4, Sutter, Novato, CA), and images were acquired at 510 nm using a cooled 12-bit digital camera (Coolsnap fx, Photometrics, Tucson, AZ) and inverted microscope (Diaphot 200, Nikon Instruments, Melville, NY) through a 20x objective. Recordings from each neuron were obtained as separate regions (MetaFluor, Molecular Devices, Downingtown, PA) at a rate of 3 Hz. After background subtraction, the fluorescence ratio R for individual neurons was determined as the intensity of emission during 340 nm excitation (I_340_) divided by I_380_, on a pixel-by-pixel basis. The Ca^2+^ concentration was then estimated by the formula K_d_·β· (R–R_min_)/(R_max_–R) where β = (I_380max_)/(I_380min_). Values of R_min_, R_max_ and β were determined by *in-situ* calibration and were 0.38, 8.49 and 9.54, and K_d_ was 224 nm [50]. Only neurons with stable baseline R traces were further evaluated. Traces were analyzed using Axograph X 1.1 (Axograph Scientific, Sydney, Australia). Sensory neurons somatic diameter is broadly associated with specific sensory modalities [16]. We therefore stratified neurons as large (> 34 μm), which represent predominantly fast conducting non-nociceptive neurons, or small (< 34 μm), which represent predominantly nociceptive neurons. Unless otherwise stated, small neurons were examined. Transients were generated by depolarization produced by microperfusion application of K^+^ (50 mM) for 0.3 s. This duration was determined in preliminary studies to generate transient amplitudes less than 400nM in most cases, to limit involvement of mitochondrial Ca^2+^ buffering [31]. Unless otherwise noted, transient measures were used only from the first depolarization following neuronal dissociation, in order to avoid amplification of PMCA function that might follow prior Ca^2+^ influx [31].

Activation of neurons by depolarization with brief bath application of K^+^ (50 mM, 0.3 s) resulted in transients that were fit well by a mono-exponential curve (Figure 1), from which the time constant (τ) was derived as a measure of the pace of recovery. PMCA function was additionally characterized by the duration of the transient from its peak to the point at which it is 95% resolved (T_95_).

### Electrophysiological recording

Small to medium size neurons were studied following dissociation, using the whole cell configuration of the patch-clamp technique at room temperature. Tyrode’s solution (see above) was used for external bath solution. The internal pipette solution contained (in mM): potassium gluconate 135, KCl 5, MgCl_2_ 2, EGTA 0.2, HEPES 10, Na_2_-phosphocreatine 10, Mg-ATP 4, Na_2_-GTP 0.3, at pH of 7.2 with KOH and osmolarity of 296 to 300 mOsm. Patch pipettes, ranging from 2-4MΩ resistance, were formed from borosilicate glass (King Precision Glass Co., Claremont, CA) and fire polished. Whole-cell recordings were made with an Axopatch 700B amplifier (Molecular Devices). Signals were filtered at 2 kHz and sampled at 10 kHz with a Digidata 1440 A digitizer and pClamp 10 software (Molecular Devices). Series resistance (7 – 14 MΩ) was monitored before and after the recordings, and data were discarded if the resistance changed by 20%. The threshold for generating APs was determined for each neuron by injecting a series of depolarizing currents with variable amplitudes lasting 1 ms. The duration of the AHP that followed a train of APs was characterized by the time constant τ determined by fitting a single exponential function of *y* = *y*_0_ + k × exp (−x/τ) (Sigmaplot 11.0, Systat Software Inc., San Jose, CA).

### Statistical analysis

Statistical analyses were performed with Statistica (StatSoft Inc, Tulsa, OK). Student’s *T*-test, Mann Whitney test, one-way ANOVA with Bonferroni’s *post-hoc* test, or repeated measures ANOVA with Dunnett’s *post-hoc* test was used to test significance of differences between groups. Where main effects were observed in ANOVA, was used to compare relevant means, and a *P* value less than 0.05 was considered significant. Data are reported as mean ± SD.

## Competing interests

The authors declared that they have no competing interests.

## Authors’ contributions

Conception and design of the experiments were performed by GG, KO, HW, and QH. Collection, analysis and interpretation of data were performed by GG, KO, BP, HW, MB, QT, and QH. Drafting the article and revising it critically for important intellectual content was performed by GG, KO, HW, and QH. All authors approved the final version of the manuscript.
